# Prophylactic Bacteriophage Administration More Effective than Post-infection Administration in Reducing *Salmonella enterica* serovar Enteritidis Shedding in Quail

**DOI:** 10.3389/fmicb.2016.01253

**Published:** 2016-08-09

**Authors:** Mosab Ahmadi, M. Amir Karimi Torshizi, Shaban Rahimi, John J. Dennehy

**Affiliations:** ^1^Department of Poultry Science, Faculty of Agriculture, Tarbiat Modares UniversityTehran, Iran; ^2^Biology Department, Queens College and The Graduate Center of the City University of New York, New YorkNY, USA

**Keywords:** phage therapy, prophylactic agent, *Salmonella* Enteritidis, phage administration method, microflora population

## Abstract

Infections caused by *Salmonella* bacteria, often through poultry products, are a serious public health issue. Because of drawbacks associated with antibiotic prophylaxis, alternative treatments are sought. Bacterial viruses (bacteriophages) may provide an effective alternative, but concerns remain with respect to bacteriophage stability and effectiveness. To this end, we assessed the stability of a novel bacteriophage isolated from poultry excreta, siphovirus PSE, and its effectiveness in reducing *Salmonella enterica* serovar Enteritidis colonization *in vitro* and *in vivo*. Moreover, we sought to determine how the timing (prophylactic or therapeutic) and route (oral gavage or vent lip) of PSE administration impacted its effectiveness. Here we report that significant quantities of viable PSE bacteriophages were recovered following exposure to high and low pH, high temperatures, and bile salts, testifying to its ability to survive extreme conditions. In addition, we found that ileal lactic acid bacteria and *Streptococcus* spp. counts increased, but colibacilli and total aerobe counts decreased, in quail receiving phage PSE through both oral gavage and vent lip routes. In other experiments, we assessed the efficiency of PSE administration, in both prophylactic and therapeutic contexts, via either oral gavage or vent lip administration, on *S.* Enteritidis colonization of quail cecal tonsils. Our results demonstrate that administration of PSE as a preventive agent could reduce the *S.* Enteritidis colonization more effectively than post-challenge administration. Furthermore, oral administration of PSE phage is a more effective prophylactic tool for reduction of *S.* Enteritidis shedding in poultry than is vent lip administration.

## Introduction

Despite impressive advances in the control of infectious diseases, some bacterial pathogens have acquired antibiotic resistance and are emerging in human populations. Many of these infections are zoonotic and are transmitted from healthy carrier animals to humans through contaminated food (Wegener et al., 2003). For example, *Salmonella* bacteria, especially the serovars Typhimurium and Enteritidis, are common contaminants of poultry and eggs, causing food-borne disease and death (Borie et al., 2008). In order to reduce pathogen contamination of the food chain and eliminate food poisoning in human population, the eradication of *Salmonella* infections before harvest and processing is crucial (Seo et al., 2000; Mølbak and Neimann, 2002). Efforts to control *S.* Enteritidis in poultry historically have relied on a combination of farm biosecurity and the prophylactic application of antibiotics, but these strategies are increasingly unreliable (Hugas and Tsigarida, 2008; Buncic and Sofos, 2012). First, indiscriminate use of antibiotics has been implicated in a surge in multidrug resistant *S.* Enteritidis. Second, poultry consumers are increasingly concerned that edible poultry tissues are contaminated with harmful concentrations of drug residues (Donoghue, 2003; Griggs and Jacob, 2005). Third, changes in food production, food rejection, and preventive measures have incurred significant economic losses to poultry producers (Tsonos et al., 2013).

Furthermore, most antibiotics, up to 90% of orally administered doses, are not fully absorbed in the chicken gut, and can be excreted in the feces unchanged (Kumar et al., 2005). As one of the largest food-producing industries in the world, the poultry industry generates enormous amounts of manure and litter, much of which is currently applied to agricultural land (Bolan et al., 2010). The effects of antibiotics contained in manure on soil microbial communities are largely unknown (Ollivier et al., 2010). In addition, poultry litter from contaminated farms may introduce zoonotic pathogens such as *S.* Enteritidis into the environment (Line and Bailey, 2006). Thus the application of poultry litter as fertilizer can create significant public and environmental health concerns. Because of these issues, there is an urgent need to find novel and effective *S.* Enteritidis control strategies to minimize the risk of spreading antimicrobial resistance among animal and human populations.

Ideally vaccination would be an optimal control strategy, but *S.* Enteritidis vaccines do not provide complete protection to infected chickens (Gast et al., 1992). There are more than 2,500 serovars of *Salmonella*, and vaccines made from any one serovar often do not confer cross-protection against others (Singh, 2009). An old strategy of using naturally occurring bacterial viruses (bacteriophages) to tackle infections, is regaining popularity (Tsonos et al., 2013). The therapeutic and prophylactic application of bacteriophages is generally considered safe (Johnson et al., 2008). Bacteriophages are highly specific to certain bacterial strains, thus presumably have minimal impact on natural human or animal microflora populations (Sulakvelidze et al., 2001). However, bacteriophage therapy has not been consistently effective (Lu and Koeris, 2011). Issues remain in terms of effective delivery routes, host resistance, limited generalizability, and possible interactions with the immune system (Loc-Carrillo and Abedon, 2011). As capsid-based entities, bacteriophages are especially sensitive to environmental conditions, such as that in the upper gastrointestinal tract. Therefore, oral administration of phages may be limited in efficiency unless the selected phages are especially resilient to environmental challenges.

An alternative method of phage administration, the vent lip of the avian cloaca, was previously described by Andreatti Filho et al. (2007). However, the relative efficacy of oral versus vent lip administration has not been comparatively assessed. In this study, a novel lytic bacteriophage against *S.* Enteritidis, PSE, was isolated and characterized in terms of pH and thermal stability, bile salts tolerance, morphology, host range, and one-step growth. In addition, the therapeutic and prophylactic effects of PSE administration on the bacterial load of quail ilea were compared. Finally, in order to provide additional insight in the feasibility of phage application as an antimicrobial agent in poultry industry, the efficacy of PSE oral versus vent lip administration to reduce *S.* Enteritidis shedding in experimentally infected Japanese quail was determined.

## Materials and Methods

### Bacterial Challenge Strain

The *Salmonella* Enteritidis (RITCC 1695) was purchased from the Razi Vaccine and Serum Research Institute (Karaj, Iran). For culture preparation, bacteria were grown in nutrient broth (Merck, Germany) at 37°C overnight. Culture turbidity was adjusted to match McFarland standard 4.

### Isolation, Purification and Enumeration of Bacteriophage

Each poultry excreta sample was collected and suspended 1:10 (w/v) in SM (salt of magnesium) buffer (50 mM Tris-HCl [pH 7.5], 0.10 M NaCl, 8 mM MgSO4⋅7H_2_O, 0.01% gelatin [Sigma, Germany]), then suspension was centrifuged at 15,610 × *g* for 20 min at 4°C. The resulting supernatant was filtered through a 0.22 μm membrane filter (Jet Biofil, China). Two hundred microliter of each filtered sample was mixed with 50 μl of fresh log-phase *S.* Enteritidis and 7 ml of top agar (0.7% agar in nutrient broth) and poured over a petri dish containing nutrient agar (1.5% agar; Serva, Germany) and incubated at 37°C for 24 h. Individual clear plaques were extracted from the agar overlay with a sterile Pasteur pipette, and suspended in 500 μl of SM buffer, which was then re-plated to obtain single plaques. Each isolate was plaque purified 4x to ensure that each isolate represented a single clone.

Phage stocks were serially diluted in SM buffer to achieve a concentration that would produce discrete plaques on a bacterial lawn. Aliquots of 200 μl of any 10 × dilution series, 50 μl of fresh log-phase *S.* Enteritidis and 7 ml of soft agar (0.7% agar in nutrient broth, 42–45°C) mixed and poured onto a plate containing nutrient agar (Serva, Germany). Plates were incubated for 24 h at 37°C and phage plaques were enumerated.

### Phage PSE Lytic Spectrum

To evaluate the lytic spectrum of isolated bacteriophages, the sensitivity of 15 bacterial strains including three *Salmonella* serovars, four *Escherichia coli* serotypes, one *Campylobacter* serotype and seven gram-positive bacteria to isolated bacteriophages was determined by spot plating phage lysates on bacterial lawns (Adams, 1959). The resulting plates were incubated overnight at 37°C, and subsequently checked for the phage plaque formation on the bacterial lawns.

### One-Step Growth

An overnight culture of *S.* Enteritidis (1 ml) was inoculated into fresh medium (100 ml) and incubated at 37°C for 1 h to yield a cell density of 1.5 × 10^8^ cfu ml^-1^. To this culture, 1 ml of isolated phage was added, giving an approximate multiplicity of infection of 0.83. Samples were taken at 5 min intervals and immediately chilled until diluting and plating for phage quantitation. Viable bacteria were counted before bacteria and phage were mixed, and subsequently assessed at intervals. Latent period was defined as the time interval between the adsorption and the beginning of the first burst, as indicated by the initial rise in bacteriophage titer. Burst size was estimated from triplicate experiments using the equation described by Jiang et al. (1998).

### Thermal and pH Stability of Bacteriophage

In order to evaluate the stability of isolated bacteriophage at various temperatures, test tubes containing bacteriophage were placed in a water bath at each temperature (30, 40, 50, 60, 70, 80, and 90°C) for 30 and 60 min (Bao et al., 2011). The surviving phages were diluted and enumerated immediately using the methods described above. In addition, the stability of bacteriophage at different pH was assessed according to the method described by Verma et al. (2009). The pH of nutrient broth was adjusted with either 1 M HCl or 1 M NaOH to obtain solutions with pH ranging from 2 to 11. A total of 1 ml of phage suspension at a titer of 10^12^ pfu ml^-1^ was inoculated into 9 ml of pH-adjusted medium to obtain a final concentration of 10^11^ pfu ml^-1^. After incubation for 2 h at 37°C, the surviving phages were diluted and counted by the methods described above. These experiments were performed three times, and the results are reported as the mean of three observation ± standard deviation.

### Bile Salts Tolerance

To determine bile salts tolerance, 1 ml of bacteriophage (10^12^ pfu ml^-1^) was placed into 9 ml of nutrient broth supplemented with 0 (control), 0.15, 0.3 and 1.0% W/V of bile salts (Quelab, Canada). After 0.5, 1, 3, and 4 h of incubation at 37°C, phage survival was assayed by diluting and enumeration of phage using the methods described above. Phage titer in each concentration of bile salts was compared with a bile salt-free control.

### Phage Morphology Examination

A drop of 10^9^ pfu ml^-1^ bacteriophage was spotted onto a formvar-coated grid and fixed for 2 min with 2.5% glutaraldehyde. Excess sample was removed, and the grid was washed with a drop of double-distilled water. Negative staining was performed by adding 1 drop of 2% uranyl acetate to the grid surface, and excess stain was removed immediately. The grid was allowed to air dry for 60 min and was then observed with a Zeiss-EM 10C transmission electron microscope (TEM) at 100 kV. Phage morphology and dimensions (head diameter, tail length) were determined by the mean of at least 5 measurements.

### Analysis of Bacteriophage Nucleic Acid

Extraction of phage nucleic acid was conducted according to the method of Binetti et al. (2005) with slight modifications. Phage particles were precipitated with 4% polyethylene glycol 6000 in 0.2 M NaCl for 48 h at 4°C and centrifuged at 15,610 × *g* for 240 min at 4°C. The pellet was re-suspended in SM buffer, supplemented with 40 μg/ml RNase A (Sinaclon, Iran) or 1 μg/ml DNase I (Sinaclon, Iran) and incubated at 37°C for 30 min. Then 80 μl of lysis solutions (0.25 M EDTA [pH 8.1], 0.5 M Tris-HCl [pH 9.6] and 2.5% sodium dodecyl sulfate [SDS]) were added to 400 μl of a concentrated suspension of phage particles, and the mixture was incubated at 65°C for 30 min. One hundred microliter of 8 M potassium acetate was then added, and the mixture was incubated on ice for 15 min before centrifugation (15,610 × *g*, 30 min, 4°C). Phage nucleic acid was precipitated from the supernatant with an equal volume of isopropanol, kept at room temperature for 5 min, then centrifuged again (15,610 × *g*, 30 min, 4°C). The pellet was re-suspended in 630 μl of TE (Tris EDTA) buffer (10 mM Tris-HCl; 1 mM EDTA, pH 8.0) in the presence of 0.3 M sodium acetate and precipitated with isopropanol for 5 min, followed by centrifugation (15,610 × *g*, 30 min, 4°C). The pellet of nucleic acid was washed twice with 70% ice-cold ethanol, dried and re-suspended in 50 μl of TE buffer.

Purified nucleic acid was digested with DNase I or RNase A according to the manufacturer’s instructions to determine nucleic acid type. We also attempted digestion with restriction enzymes *Eco*RI and *Hin*dIII according to the manufacturer’s instruction (Serva, Germany). Products of digested phage nucleic acid were electrophoresed by 0.8% agarose gel in a TAE (Tris Acetate EDTA) buffer (40 mM Tris-acetate, 1 mM EDTA) and visualized by transillumination with UV light after the gels were stained with ethidium bromide (Serva, Germany).

### Determination of Phage Genome Size by Agarose Gel Electrophoresis

The relatively small genome of PSE made it possible to use agarose gel electrophoresis for determination of its size. The distance of PSE DNA migration on agarose gel electrophoresis was compared to that of un-cut and *Hin*dIII lambda DNA fragments as DNA size marker by Photo Capt software version 12.4 (Vilber Lourmat, France) (Brown, 2010).

### Experimental Analysis of Bacteriophage Treatment on *S.* Enteritidis Infection

This project maintained proper ethical standards and all experiments were approved by the Animal Care Committee of Tarbiat Modares University.

#### Experiment 1

To evaluate the effects of isolated phages on bird natural microflora, ninety 33-day-old *Salmonella*-free quail were randomly divided into three groups of 30 birds. All birds received an identical diet, and feed and water were supplied *ad libitum*. A control group (Group 1) did not receive bacteriophage treatment. Group 2 received 10^9^ pfu ml^-1^ bacteriophage in 100 μl aliquot by oral gavage for 2 days, once every 24 h and group 3 received 10 μl of 10^10^ pfu ml^-1^ bacteriophage, via vent lip route for 2 days, once every 24 h.

Bacterial quantitation of the ileal contents of 10 birds per each group was done at 36 days. One gram of the ileal contents of each bird was serially diluted in phosphate-buffered saline from 10^-1^ to 10^-6^. Dilutions were subsequently plated on selective agar media for enumeration of target bacteria. Lactic acid bacteria, colibacilli, streptococci and total aerobes were plated on de Man, Rogosa and Sharpe agar (MRS, Merck, Germany), Mac Conkey agar (Merck, Germany), Kenner Fecal *Streptococcus* agar (KF *Streptococcus*, Merck, Germany), and plate count agar (Merck, Germany), respectively. Plates were incubated at 37°C overnight, and then the resulting colonies were counted.

#### Experiment 2

To compare the efficiency of prophylactic versus therapeutic application of phage on *S.* Enteritidis shedding, one hundred 8-day-old *Salmonella*-free quail were randomly divided into four groups of 25 birds. Group 1 (prophylactic treatment) received 100 μl of 10^6^ pfu ml^-1^ bacteriophage via oral gavage for 3 days, once every 24 h, before being challenged orally by 100 μl of 1.2 × 10^9^ cfu ml^-1^ *S.* Enteritidis (**Table 1**). Group 2 (therapeutic treatment) received 100 μl of 1.2 × 10^9^ cfu ml^-1^ *S.* Enteritidis first, then received bacteriophage as described in group 1 (**Table 1**). Birds of group 3 were challenged like group 2, but did not receive phage treatment (**Table 1**). Neither *Salmonella* nor bacteriophage was administered to group 4 (**Table 1**).

**Table 1:** Scheme of Experiment 2.

Day^a^	Group 1 (preventive)		Group 2 (therapeutic)		Group 3 (positive)	Group 4 (negative)
				
	Phage dose^b^	SE dose^c^	Phage dose^b^	SE dose^c^	SE dose^c^	–
8	10^6^	–	–	1.2 × 10^9^	1.2 × 10^9^	–
9	10^6^	–	10^6^	–	–	–
10	10^6^	–	10^6^	–	–	–
11	–	1.2 × 10^9^	10^6^	–	–	–


*^a^Day of hatching is given as day 0. ^b^Dose of phage given as pfu ml^-*1*^. ^c^Dose of *S.* Enteritidis given as cfu ml^-*1*^.*

The presence of *S.* Enteritidis was assayed at 12, 24 h, and 7 days following *S.* Enteritidis challenge. Birds were euthanized by CO_2_ inhalation, and their cecal tonsils were aseptically removed, homogenized and incubated overnight at 37°C in 10 ml of peptone water buffer (Merck, Germany). Subsequently, 100 μl of cultured peptone water was transferred into Rappaport–Vassiliadis broth (Merck, Germany) at 37°C for 24 h, and then streaked on xylose-lysine desoxycholate (XLD) agar plates (Merck, Germany). Plates were incubated at 37°C for 24 h. Any black colonies that formed were identified as *Salmonella* Enteritidis. Any doubtful colonies were plated on triple sugar iron agar (TSI agar, Merck, Germany) slants. In addition, urease activity was checked by assaying the degradation of urea in urea broth (Stager et al., 1983). The recovery of *S.* Enteritidis is reported as the number of *S.* Enteritidis positive samples per number of total samples.

#### Experiment 3

The effectiveness of oral gavage versus vent lip administration of phage on *S.* Enteritidis colonization in cecal tonsils of quail was evaluated in a third experiment. Three hundred, 1-day-old *Salmonella*-free quail were randomly assigned to five groups of 60 birds. Each group of birds was placed in cages in a controlled environment under strict biosecure conditions. Fecal samples were taken to determine if any pre-existing *Salmonella* phages were present using the method described above. Feed and water were provided *ad libitum*, and birds were maintained at an age-appropriate temperature for the duration of the experiment.

Neither *Salmonella* nor bacteriophages were administered in group 1. Group 2 birds did not receive bacteriophage, but at the age of 4 days were challenged orally by 100 μl of 1.2 × 10^9^ cfu ml^-1^ *S.* Enteritidis. Birds in group 3 received 100 μl of 10^9^ pfu ml^-1^ bacteriophage via oral gavage every 24 h for 6 days, starting 3 days before *S.* Enteritidis challenge. Group 4 received 5 μl of 2 × 10^10^ pfu ml^-1^ bacteriophage, via the vent lip route every 24 h for 6 days, starting 3 days before *S.* Enteritidis challenge. Birds of group 5 received bacteriophages similar to groups 3 and 4, but at half the concentration, and were not challenged with *S.* Enteritidis (**Table 2**). Necropsies of birds from each group were performed at 6, 12 h, 1, 2, 3, 7, 14, 28, and 35 days post-challenge. The presence of *S.* Enteritidis was assayed as described above. In this experiment, recovery of bacteriophage from bird feces was done by using described method for phage isolation.

**Table 2:** Scheme of Experiment 3.

Day^a^	Group 1 (negative control)	Group 2 (positive control)	Group 3 (oral gavage)		Group 4 (vent lip)		Group 5 (phage control)	
					
		SE dose^c^	Phage dose^b^	SE dose^c^	Phage dose^b^	SE dose^c^	Oral	Vent
1	^-^	^-^	10^9^	^-^	2 × 10^10^	^-^	10^9^	2 × 10^10^
2	^-^	^-^	10^9^	^-^	2 × 10^10^	^-^	10^9^	2 × 10^10^
3	^-^	^-^	10^9^	^-^	2 × 10^10^	^-^	10^9^	2 × 10^10^
4	^-^	1.2 × 10^9^	10^9^	1.2 × 10^9^	2 × 10^10^	1.2 × 10^9^	10^9^	2 × 10^10^
5	^-^	^-^	10^9^	^-^	2 × 10^10^	^-^	10^9^	2 × 10^10^
6	^-^	^-^	10^9^	^-^	2 × 10^10^	^-^	10^9^	2 × 10^10^


*^a^ Day of hatching is given as day 0. ^b^ Dose of phage given as pfu ml^-1^. ^c^Dose of *S.* Enteritidis given as cfu ml^-1^.*

### Statistical Analysis

The data related to the bacterial enumeration of quail ileal contents were compared between groups using a generalized linear model (GLM) implemented in SAS with subsequent Duncan’s multiple range test (SAS Institute, 2003). The significance of differences in the incidence of *S.* Enteritidis recovery between control and phage-treated experimental groups was determined by the chi-square test (*p* **≤** 0.05).

## Results

### Phage PSE Isolation and Lytic Spectrum Assay

**FIGURE 1:**
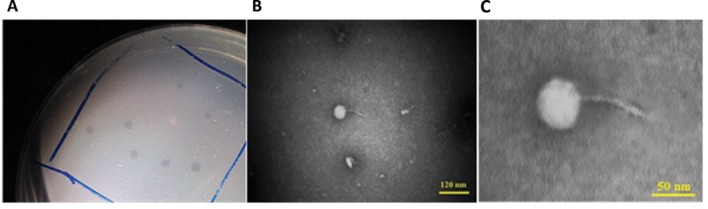
**Associated plaque **(A)** and transmission electron micrographs of PSE phage under 100 kV magnification.** The bars represent 120 nm **(B)** and 50 nm **(C)**.

One *Salmonella*-lysing phage, named PSE, was isolated from poultry’s excreta using an enrichment strategy (Cross et al., 2015). PSE forms clear 2–3 mm plaques on *Salmonella* Enteritidis (**Figure 1A**). A lytic spectrum test indicated that PSE was able to lyse three strains of *Salmonellae*, but was unable to lyse bacteria of other genera (**Table 3**).

**Table 3:** The lytic spectrum of PSE on 15 bacterial strains from 7 genera.

Strain	Sources/Reference	Lysis by bacteriophage PSE
*Salmonella* Enteritidis RITCC 1695	RVSRI^1^	+
*Salmonella* Typhimurium	Faculty of Veterinary, Tehran University	+
*Salmonella* Pullorum RITCC 1818	RVSRI^1^	+
*Campylobacter jejuni* RITCC 1097	RVSRI^1^	-
*Escherichia coli* O1:K1	RVSRI^1^	-
*Escherichia coli* O2:K1	RVSRI^1^	-
*Escherichia coli* O78:K80	RVSRI^1^	-
*Escherichia coli* Nissle 1819	Isolated from Mutaflor^®^, Germany	-
*Lactobacillus rhamnosus* TMU094	Karimi Torshizi et al., 2008	-
*Lactobacillus fermentum* TMU121	Karimi Torshizi et al., 2008	-
*Pedioccus pentosaceus* TMU457	Karimi Torshizi et al., 2008	-
*Pediococcus acidilactici*	Isolated from Bactocell^®^, France	-
*Enterococcus faecalis* ATCC 51299	ATCC^2^	-
*Enterococcus faecalis* ATCC 19433	ATCC^2^	-
*Bacillus subtilis*	Isolated from Gallipro^®^, Denmark	-


*^1^Razi vaccine and serum research institute, Iran. ^2^American Type Culture Collection, USA.*

### Phage PSE Morphology

Phage PSE has a round head with a diameter of 51.29 ± 3.15 nm and a tail with length of 74.53 ± 0.74 nm. From its morphology, PSE was presumptively identified as a member of the *Siphoviridae* family with long non-contractile tail in the order of *Caudovirales* (**Figures 1B,C**).

### Phage PSE One-Step Growth Dynamics

**FIGURE 2:**
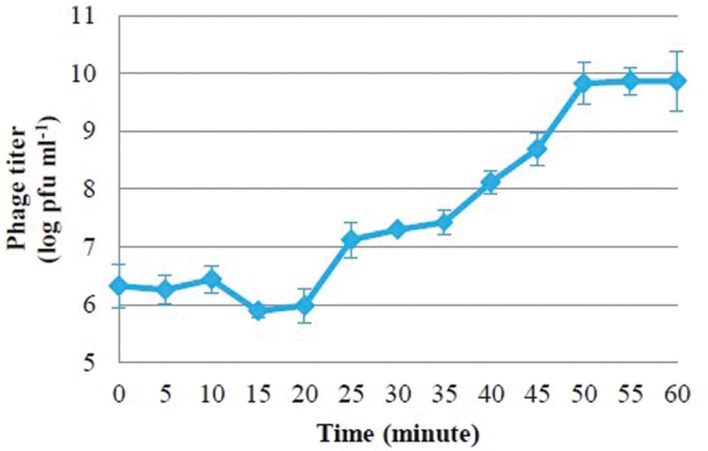
**One-step growth curves of bacteriophage PSE in *Salmonella* Enteritidis**.

One-step growth curves of PSE were conducted to determine the growth pattern and the number of progeny phages released by lysing a single bacterial host cell. One-step growth curves for phage PSE showed a latent period and burst size of about 20 min and 66.66 ± 6.67 pfu cell^-1^, respectively (**Figure 2**).

### Phage PSE Stability at Different Bile Salts Concentrations, Temperatures and pH

**FIGURE 3:**
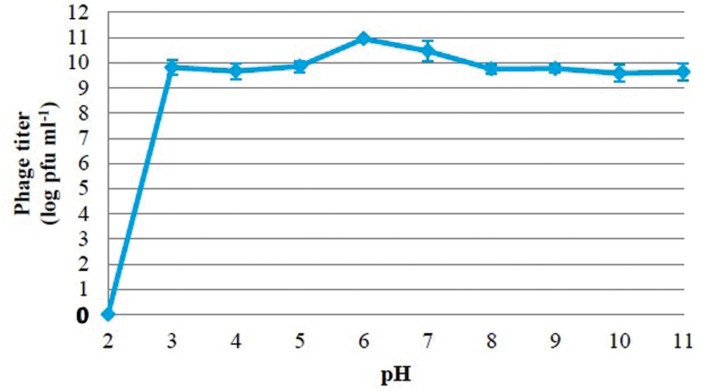
**Effect of pH on stability of phage PSE**.

**FIGURE 4:**
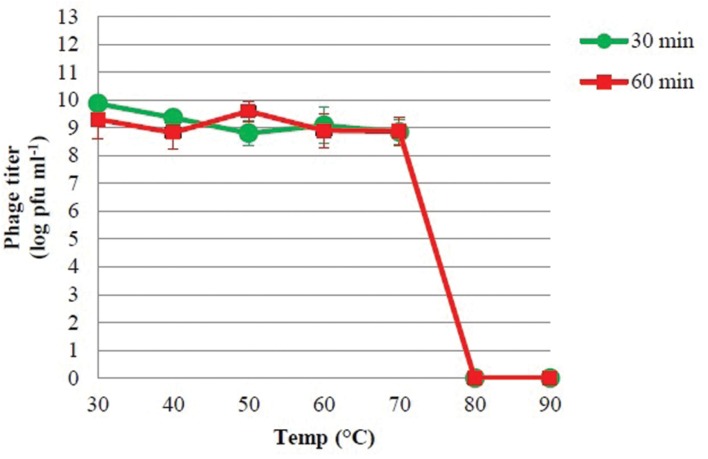
**The thermal stability of phage PSE**.

The viability of phage PSE was fully maintained and no reduction in PSE titer was observed after 4 h of exposure to any of the tested bile salts concentrations (data not shown). No obvious effect on PSE activity was observed after 2 h incubation at pH levels ranging from 3 to 11 (**Figure 3**). In addition, the results of thermal stability tests suggested that phage PSE was relatively heat stable up to 60 min at temperatures between 30°C to 70 °C. At 80°C or higher, the phage titer quickly dropped, no viral particles were detected after 30 min of incubation, and phage activity was completely lost (**Figure 4**).

### Phage PSE Genomic Characterization

**FIGURE 5:**
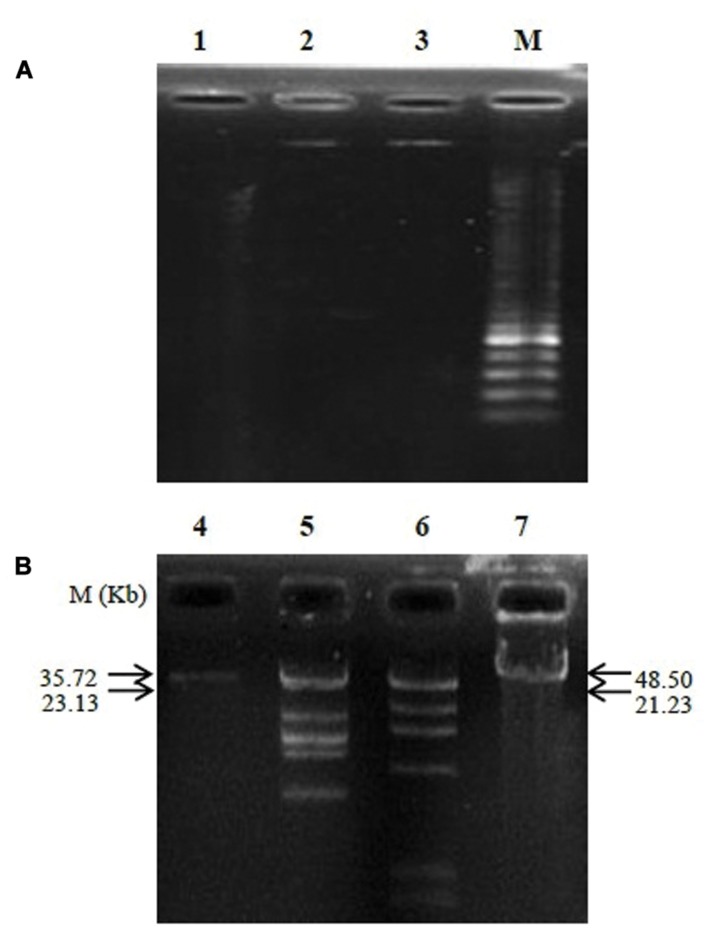
**Agarose gel electrophoresis analysis of genomic nucleic digested with DNase I and RNase A **(A)** and determination of phage genome size **(B)**.** Lanes contained the following: 1, digested genome of PSE with DNase I; 2, un-cut genome of PSE with RNase A; 3 and 4, PSE genome; 5, *Hin*dIII + lambda DNA; 6, *Eco*RI + lambda DNA; 7, lambda DNA; M, molecular weight marker.

The analysis of phage nucleic acid suggested that PSE is a DNA phage as the genome was completely digested by DNase I, but refractory to the activities of RNase A. The estimated genome size of PSE was approximately 35.72 kb (**Figure 5**). Two restriction enzymes used in this study, *Eco*RI and *Hin*dIII, were unable to digest the PSE genome.

### Effect of Phage PSE on Bacterial Frequencies in the Quail Ilea

We assessed the impact of phage PSE administration on quail ileal bacteria. Our results show that lactic acid bacteria and streptococci counts increased relative to controls when PSE was administered via oral gavage and vent lip methods (**Table 4**; *P* = 0.0001). By contrast, colibacilli and total aerobes counts decreased relative to controls following both treatment methods (**Table 4**; *P* = 0.0001 and 0.005 respectively). Administration of bacteriophage PSE via oral gavage and vent lip routes increased lactic acid bacteria count by 1.47 and 0.73 log cfu g^-1^, respectively (**Table 4**). Also streptococci counts increased by 1.05 and 0.58 log cfu g^-1^, when PSE was administered by oral gavage and vent lip routes, respectively (**Table 4**).

**Table 4:** Effect of phage PSE treatment on bacterial counts in quail ilea (Log _10_ CFU g^-1^ ileal contents, mean of 10 birds per each group).

Groups	Bacterial group			
	
	Total aerobes	Colibacilli	Lactobacilli	Streptococci
1 (Control)	8.54	8.31	6.31	5.74
2 (Oral)	7.39	7.99*^ns^*	7.78	6.79
3 (Vent lip)	7.79	7.84*^ns^*	7.04	6.32
SEM^∗^	0.008	0.01	0.001	0.003
*P-*value^∗^	0.0001	0.005	0.0001	0.0001


*^∗^SEM and *P*-values were calculated using a GLM univariate analysis of variance. *Post hoc* comparisons (Duncan’s test) of mean values within each column revealed that all are significantly different (*P*-value < 0.05) from other groups in the same column except the pair labeled with the superscript *ns*.*

### Comparison of Preventive versus Therapeutic Phage Treatment

We tested the effectiveness of PSE treatment, either prophylactically prior to *S.* Enteritidis exposure or therapeutically after *S.* Enteritidis exposure. One day post-challenge, *S.* Enteritidis was isolated from 100% of cecal tonsils of positive control and therapeutic groups. By contrast, *S.* Enteritidis isolation rates in birds of negative control and preventive groups were 0 and 33.3% respectively (**Table 5**). In addition, 7 days post-challenge, *S.* Enteritidis was isolated from 100% of positive control and therapeutic groups (**Table 5**). By contrast, the *S.* Enteritidis isolation rate from the preventive group was 20% and birds of the negative control remained free from *S.* Enteritidis.

**Table 5:** Frequency of *S.* Enteritidis positive cecal tonsils per group (%).

Groups	Time of sampling		
	
	12 h^1^ post-challenge	24 h^1^ post-challenge	7 d^2^ post-challenge
1 (Prophylactic)	2/6 (33.33%)	2/6 (33.33%)	2/10 (20%)
2 (Therapeutic)	6/6 (100%)	6/6 (100%)	10/10 (100%)
3 (Positive control)	6/6 (100%)	6/6 (100%)	10/10 (100%)
4 (Negative control)	0/6 (0%)	0/6 (0%)	0/10 (0%)


*^1^Hour. ^2^ Day.*

### Comparison of Oral versus Vent Lip Administration of Phage PSE

The application of phage PSE, either by oral or vent lip routes, reduced *S.* Enteritidis recoveries from cecal tonsils of quail in nine consecutive samplings (**Table 6**). Administration of PSE via the oral route was able to eliminate *S.* Enteritidis at all sampling times except 6 h post-challenge. One week after treatment, *S.* Enteritidis was recovered from 100% of cecal tonsil samples of the positive control group, while no *S.* Enteritidis was found in birds that orally received bacteriophage PSE (**Table 6**). Overall, at all sampling times, *S.* Enteritidis was isolated from 90.74% of cecal tonsil samples of the positive control group. By contrast, *S.* Enteritidis isolation rates in birds treated with bacteriophage PSE via oral and vent lip routes, and in the negative control, were 2.77, 17.58, and 0% respectively (**Table 6**). Bacteriophages were recovered from fecal specimens of all bacteriophage-receiving groups, regardless of administration routes (**Table 7**).

**Table 6:** Efficiency of phage PSE administration methods on *S.* Enteritidis recovery from cecal tonsils of Japanese quail (*S.* Enteritidis positive cecal tonsil/ total).

Groups	Time post-challenge						
	
	6 h^1^	12 h	1 d^2^	2 d	3 d	7 d	14 d	28 d	35 d
1 (Negative)	0/8	0/6	0/8	0/6	0/6	0/6	0/6	0/6	0/8
2 (Positive)	6/8	6/6	6/8	4/6	6/6	6/6	6/6	6/6	8/8
3 (Oral)	2/8	0/6	0/8	0/6	0/6	0/6	0/6	0/6	0/8
4 (Vent lip)	2/8	2/6	0/8	0/6	2/6	0/6	2/6	2/6	0/8
5 (Phage)	0/8	0/6	0/8	0/6	0/6	0/6	0/6	0/6	0/8


*^1^Hour. ^2^ Day.*

**Table 7:** Recovery of bacteriophage from feces of birds after 7 days post treatment.

Groups	Bacteriophage (Log pfu/g)^a^
1 (Negative control)	0
2 (Positive control)	0
3 (Oral gavage)	10^5^
4 (Vent lip)	10^4^
5 (Phage control)	10^5^


*^a^Average of 10 birds.*

## Discussion

A rise in antibiotic-resistant *S.* Enteritidis bacteria, and concerns about the ecological and environmental effects of unrestricted antibiotic usage, have provided strong motivation to find new and effective prophylactic and therapeutic means of eradicating *S.* Enteritidis from poultry. Bacteriophages have excellent potential for replacing or supplementing antibiotics, but their effectiveness and stability must be demonstrated experimentally. Here we describe the isolation, characteristics and effectiveness of a novel bacteriophage able to infect *S.* Enteritidis.

On the assumption that phages isolated from poultry’s excreta are more stable in the poultry gastrointestinal tract compared to isolated bacteriophages from other sources, we isolated a bacteriophage, which we called PSE, from poultry excreta. Bacteriophage PSE has a number of characteristics that make it a strong phage therapy candidate. Bacteriophage PSE formed 2–3 mm round, clear plaques on *S.* Enteritidis, implying that it is a lytic phage (Yoon et al., 2007). Because they quickly reproduce within and lyse host bacteria, lytic phages are more suitable than lysogenic phages for phage therapy (Abedon, 2008). Moreover, although PSE was isolated using *S.* Enteritidis, it was also able to infect and lyse *S*. Typhimurium and *S*. Pullorum, two other significant bacterial pathogens found in poultry (**Table 3**).

Bacteriophage PSE’s latent period was estimated to be about 20 min, and its burst size was approximately 66.66 ± 6.67 pfu cell^-1^. There seems to be considerable variation in latent period length and burst size among *Salmonella*-specific phages. Some *Salmonella*-specific phages have been reported to possess small burst sizes and long latent periods, for example *ϕ st*1 (22 pfu cell^-1^, 40 min) (Wong et al., 2014) and phage Felix 01 (14 pfu cell^-1^, 60 min) (O’Flynn et al., 2006). On the other hand, some *Salmonella*-specific phages were isolated with burst sizes range from 100 to 200 pfu cell^-1^ and shorter latent periods (Ahiwale et al., 2013). Differences in medium, host cell, pH and temperature may affect variations in latent period and burst size of different phage isolates (Müller-Merbach et al., 2007). For the purposes of phage therapy, optimum latent periods and burst sizes have not been established; however, there is some evidence that these parameters can vary considerably depending on host density and physiological status (Abedon, 1989; Abedon et al., 2001; Wang, 2006).

Phages used for therapeutic or prophylactic purposes should be highly stable and remain viable in a wide range of potential environments. Phage PSE meets some of these requirements. It exhibited powerful antibacterial activity at temperatures ranging from 30°C to 70°C, and pH levels ranging from 3 to 11. Also PSE was found to be resistant to high bile salt concentrations, remaining viable even after 4 h of exposure to 1% bile solution. These characteristics suggest that phage PSE would remain therapeutically viable inside animal bodies, including the animal gastrointestinal tract, where *Salmonella* may be prevalent. All of these features point to the utility of phage PSE as a stable agent for control of *S.* Enteritidis colonization of poultry.

In the intestine, bacterial populations can interact with each other, and phages are expected to have a significant role in driving the biodiversity of this complex ecosystem (Ventura et al., 2011). Our data demonstrate that the PSE phage administration, either by oral or vent lip method, strongly affected ileal bacteria frequencies (**Table 4**). We observed lower frequencies of coliforms in phage-treated birds compared with controls (**Table 4**). The declines in coliforms were matched by increases in lactic acid bacteria and streptococci (**Table 4**). *Lactobacillus* spp. are often thought to have a positive effect on poultry health by reducing or preventing colonization of the poultry intestinal tract by undesirable bacteria (Karimi Torshizi et al., 2008). However, given the complexities of the gut microbiome, it is difficult to determine the direct mechanism by which PSE treatment may have influenced the relative numbers of coliforms, lactic acid bacteria and streptococci. This finding is nonetheless an intriguing topic for further investigation.

The *S.* Enteritidis reductions observed in cecal tonsil samples following preventive treatment strongly suggest that phage PSE is a viable prophylactic against *S.* Enteritidis colonization (**Table 5**). It is commonly believed that bacteriophages administered to treated animals are present for the duration of the infection, but once the bacterial host is eliminated so too is the bacteriophage. In our experiments, this did not happen. Bacteriophage PSE was isolated from feces of all groups that received it, even after *S.* Enteritidis infection was no longer observed. Interestingly, phage PSE persistence was also observed in the absence of *S.* Enteritidis challenge (**Table 7**). This finding suggests that phage PSE either remains inactive in gastrointestinal tract for long periods or may have alternate hosts that allow it to proliferate. Similarly, it was reported that, in the absence the primary host, the bacteriophage UZ1 persisted 13 days in a simulated colon (Verthé et al., 2004). This evidence lends credibility to the prophylactic ability of PSE.

This result is in sharp contrast to the observation that the therapeutic PSE treatment of birds infected by *S.* Enteritidis failed to eradicate *S.* Enteritidis from bird cecal tonsils relative to controls (**Table 5**). It may be that PSE treatment of extant infections rapidly selected for PSE resistance in *S.* Enteritidis bacteria or that *S.* Enteritidis that had previously colonized the treatment animals were resistant to infection (Higgins et al., 2008). This experiment highlights the importance of phage administration as a prophylactic prior to *S.* Enteritidis infection is a more effective strategy than phage administration as a treatment following *S.* Enteritidis infection (**Table 5**). Presumably, prophylactic PSE treatment can prevent establishment of *S.* Enteritidis infection, but is relatively ineffective against established *S.* Enteritidis infections.

In another experiment, phage PSE was applied to treated birds, either orally or via the vent lip, for 3 days prior to infection with *S.* Enteritidis. Following *S.* Enteritidis challenge, all birds were tested for *S.* Enteritidis infection periodically across 35 days. In birds treated with PSE orally, *S.* Enteritidis was detected in the cecal tonsils 6 h following *S.* Enteritidis challenge, but not subsequently (**Table 6**). Some birds treated with phage PSE via the vent lip periodically tested positive for *S.* Enteritidis across the study period, but the majority remained free of *S.* Enteritidis (**Table 6**). Birds in the negative control (no treatments) and birds receiving only bacteriophage treatment remained free from contamination with *S.* Enteritidis across the entire study period (**Table 6**). These results imply that the bird quarantine was effective and no cross contamination occurred during the entire experimental period. Significantly, while both oral and vent lip treatment routes reduced *S.* Enteritidis infection compared to the positive control, the oral treatment route was more effective than the vent lip method in treatment of *S.* Enteritidis infections.

Although some studies suggested that it might be necessary to employ cocktails of bacteriophages to provide protection against *S.* Enteritidis (Atterbury et al., 2007; Borie et al., 2008), our study demonstrates that treatment with a single phage type can effectively prevent *S.* Enteritidis colonization. For practical and economic reasons, employing a single phage to achieve therapeutic effect is more desirable than cocktails that contain several types of phage (Sulakvelidze et al., 2001).

## Conclusion

Phage PSE shows great promise for the prevention and treatment of *S.* Enteritidis infection, and it may be a plausible alternative to antibiotics for the reduction of *S.* Enteritidis shedding in poultry. Phage PSE was most effective when administered prophylactically prior to *S.* Enteritidis infection than as a treatment for established *S.* Enteritidis infections. Although we observed a reduction in *S.* Enteritidis infection in birds prophylactically treated with phage PSE via the vent route, our results indicate that administration of phage PSE via oral route is most effective.

## Highlights

Phage PSE persistence was observed in the absence of *S.* Enteritidis challenge. Administration of phage PSE as a preventive agent could reduce the *S.* Enteritidis colonization more effectively than post challenge administration. Administration of phage PSE via oral route is most effective for reducing *S.* Enteritidis colonization.

## Author Contributions

MA and MK designed the study, performed experiments, analyzed the data and wrote the manuscript. SR and JD reviewed the manuscript. All authors read and approved the final manuscript.

## Conflict of Interest Statement

The authors declare that the research was conducted in the absence of any commercial or financial relationships that could be construed as a potential conflict of interest.
